# *Ambengana* Millidge & Russell-Smith, 1992, a synonym of *Neriene* Blackwall, 1833 (Araneae, Linyphiidae)

**DOI:** 10.3897/zookeys.52.496

**Published:** 2010-07-30

**Authors:** Xin Xu, Liu Jie, Jian Chen

**Affiliations:** College of Life Sciences, Hubei University, Wuhan 430062, Hubei, China; College of Life Sciences, Hubei University, Wuhan 430062, Hubei, China; College of Life Sciences, Hubei University, Wuhan 430062, Hubei, China

**Keywords:** Taxonomy, Linyphiidae, Neriene, Ambengana, new synonymy

## Abstract

The taxonomic status of the genus Ambengana Millidge & Russell-Smith, 1992, is revised on the basis of its original description, illustrations and re-examination of the type species. A new synonymy is proposed: Ambengana complexipalpis Millidge & Russell-Smith, 1992 (the type species of Ambengana) **syn. n.** with Neriene birmanica (Thorell, 1887). Therefore,the genus Ambengana Millidge & Russell-Smith, 1992 **syn. n.** is synonymized with Neriene Blackwall, 1833. A morphological re-description, diagnosis and comparative illustrations are provided for Neriene birmanica as well.

## Introduction

The monotypic genus Ambengana Millidge & Russell-Smith, 1992, was erected for Ambengana complexipalpis Millidge & Russell-Smith, 1992, from Bali (Millidge and Russell-Smith 1992). There has been no further report on this genus since its original description (Platnick 2010). Recently, the authors examined the linyphiid specimens collected from Yunnan Province in the southwestern China and found that some of them seemed to be Ambengana complexipalpis. However, the structure of the copulatory organs of this species clearly indicated that this species should be considered a junior synonym of Neriene birmanica (Thorell, 1887). The spermathecae with spirally coiled grooves in the female, the spirally coiled terminal apophysis, and the curved and narrow embolus in the male, all indicate that this species should probably belong to the genus Neriene. Thus, the genus Ambengana should be considered a junior synonym of the genus Neriene Blackwall, 1833.

Neriene birmanica was first described as Linyphia birmanica from the female (Thorell, 1887). Since then several studies have been made (Caporiacco 1935; van Helsdingen 1969; Chen et al. 1989; Chen and Gao 1990; Song et al. 1999). Among these, Caporiacco (1935) first reported the male as Bathyphantes kashmiricus in Kashmir; van Helsdingen (1969) redescribed the male as Neriene kashmirica when he reclassified the species of Linyphia latreille; Chen et al. (1989) first reported on this species from China and synonymized Neriene kashmirica with Neriene birmanica. The aim of the current paper is to re-describe Neriene birmanica, and to illustrate with digital photos, and to propose a new synonymy.

## Material and methods

Specimens were examined with an Olympus SZX16 stereomicroscope; details were studied with an Olympus BX51 compound microscope. Male palps and female epigyne were examined and illustrated after being dissected from the spider bodies. Spermathecae were cleared in boiling KOH solution to dissolve soft tissues, and the embolic divisions of male palps were excised by breaking the column (the membranous connection between the suprategulum and the radix). Photos were made with Cannon G10 digital camera (14.7 megapixels) mounted on an Olympus SZX16 dissecting microscope. The digital images depicting the general appearance and genital morphology are a composite of multiple images taken at different focal lengths along the Z axis and assembled using the software package Helifocus 3.10. Left structures (e.g., palps, legs, etc.) are depicted unless otherwise stated. Most hairs and macrosetae are usually not depicted in the final palp and epigynum images.

All measurements were obtained using an Olympus SZX16 stereomicroscope and are given in millimeters. Eye diameters are taken at the widest point. The total body length does not include the length of the chelicerae or spinnerets. The leg measurements are given in the following sequence: total (femur, patella +tibia, metatarsus, tarsus). The terminology used in text and figure legends follows van Helsdingen (1969).

The following abbreviations are used in the text and figures. Male palp: ALPanterior projection of lamella; DLPdorsal projection of lamella; EEmbolus; MMmedian membrane; MA-median apophysis; Llamella; LLPlateral projection of lamella; Pparacymbium; PLPposterior projection of lamella; STsubtegulum; Ttegulum; TAterminal apophysis. Epigynum: FGfertilization groove; Sspermatheca; SC“scape”; SGspiral groove; TPturning point. Somatic characters: AERanterior eye row; ALEanterior lateral eye; AMEanterior median eye; AME-ALEdistance between AME and ALE; AME-AMEdistance between AMEs; AMEddiameter of AME; PERposterior eye row; PLEposterior lateral eye; PMEposterior median eye; PMEddiameter of PME; PME-PLEdistance between PME and PLE; PME-PMEdistance between PMEs.

All the specimens examined are deposited in the College of Life Sciences, Hubei University, China.

## Taxonomy

### Neriene

Blackwall, 1833

#### Type species.

Linyphia clathrata Sundevall, 1830.

#### Neriene birmanica

(Thorell, 1887)

Figs 1 12

Linyphia birmanica Thorell, 1887: 99 (f).
Bathyphantes kashmiricus Caporiacco, 1935: 167, pl. 2, fig. 12 (m).
Neriene kashmirica van Helsdingen, 1969: 261, figs 359–360 (m).
Neriene birmanica van Helsdingen, 1969: 265, figs 361–363 (f).
Neriene birmanica Chen, Zhu & Chen, 1989: 1, figs 1–10 (mf).
Neriene birmanica Chen, Gao, 1990: 99, figs 124a–j (mf).
Neriene birmanica Song, Zhu & Chen, 1999: 188, figs 108G–H, Q (mf).
Ambengana complexipalpis Millidge, Russell-Smith, 1992: 1387, figs 52–55 (mf), **syn. n.**

##### Material examined.

CHINA: 1 ♂, 3 ♀, Sichuan Province, Panzhihua City, Miyi County, 10.07.1981 Gao J.C.; 1 ♂, 1 ♀, same locality, Chen X.E.; 2 ♂, 3 ♀, Yunnan Province, Dehong DaiJingpo Autonomous Prefecture, Ruili City, 16.09.2000, Chen, W. H. and Liu, F. X.; 2 ♂, 6 ♀, Yunnan Province, Lincang City, Zhenkang County, Nansan Town, 14.09.2000, Chen, W. H. and Liu, F. X.; 1 ♀, Yunnan Province, Honghe Hani and Yi Prefecture, Luchun County, 2.09.2000, Chen, W. H. and Liu, F. X.; 3 ♂, 7 ♀, Yunnan Province, Xishuangbanna Dai Autonomous Prefecture, Menghai County, Daluo Town, 9.09.2000, Chen, W. H. and Liu, F. X.; 1 ♂, same province and prefercture, Menghai County, 10.09.2000, Chen, W. H. and Liu, F. X.; 1 ♀, Yunnan Province, Honghe Hani and Yi Prefecture, Jinping Miao, Yao and Dai Autonomous County, 30.08.2000, Chen, W. H. and Liu, F. X.; 1 ♀, Yunnan Province, Pu'er Prefecture, Jiangcheng Hani and Yi Autonomous County, 25.09.2000, Chen, W. H. and Liu, F. X.

**Figures 1–2.**
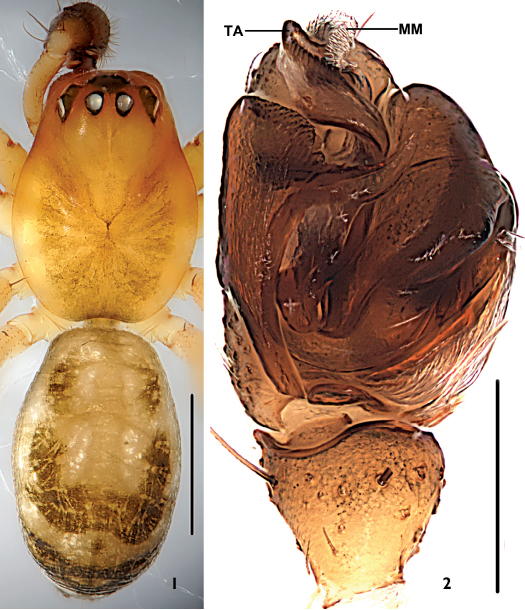
Neriene birmanica (Thorell, 1887), male from Nansan Town (Yunnan Province, China). **1** male habitus, dorsal view **2** left palp, ventral view. Scale bars =1 mm (**1**), scale = 0.2 mm (**2**).

##### Diagnosis.

Tibia not fusiform, with few spines; paracymbium with narrow distal arm tapering to a sharp tip; hook-shaped tip of distal part of median apophysis curved in ventral direction; transversal and terminal sclerites never present; lateral depressions of the epigyne small and superficial, this species belongs to the peltata-species group (van Helsdingen 1969). It can be distinguished from other members of the peltata-group species by the tiny paracymbium (Fig. 4), the sword-shaped embolic tip (Figs 4, 6), the broad and short terminal apophysis with about one coil (Figs 2, 5), the translucent spot at either side of the uniquely trapeziform atrium opening, the superficially depressed area at either side next to lateral translucent spots (Fig. 9), and the scape forming a rounded mesal projection and spiral grooves of about two coils in the female (Figs 10, 11).

##### Description.

###### Male:

Total length: 2.78. Carapace: 1.22 long, 0.94 wide. Abdomen: 1.54 long, 0.98 wide. Carapace brown, unmodified. Eyes subequal. AER recurved, AME-AME shorter than AMEd, AME-ALE slightly longer than AMEd; PER straight, PME-PME about PMEd, PME-PLE slightly longer than PMEd; ALE and PLE juxtaposed. Chelicerae brown, stridulatory ridges absent, promargin of fang groove with three teeth, median tooth largest;, retromargin with three small teeth, first bigger than others. Lengths of legs: I 5.36 (1.47+1.58+1.52+0.79), II 4.90 (1.33+1.43+1.38+0.76), III 4.08 (1.19+1.16+1.08+0.65), IV 4.41 (1.25+1.21+1.29+0.66). Each tibia, patella and femur with two dorsal spines. Tm I: 0.20. Tm IV absent. Abdomen cylindriform, without tubercle; colour and pattern as in Fig. 1.

**Figures 3–7.**
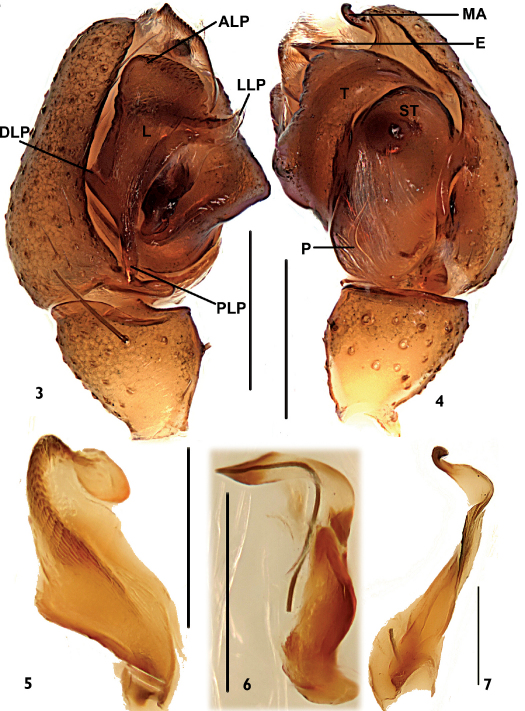
Neriene birmanica (Thorell, 1887), male from Nansan Town (Yunnan Province, China). **3** left palp, prolatral view **4** left palp, retrolateral view **5** terminal apophysis, retrolateral view **6** embolus, retrolateral view **7** median apophysis, retrolateral view. Scale bars = 0.2 mm.

Patella short, with long spine dorsally. Tibia shorter than cymbium, with several long spines on lateral and ventral surfaces, and one prodorsal, two retrodorsal trichobothria. Paracymbium tiny, long and slender, U-shaped, slightly membranous (Fig. 4). Median apophysis long, slender in lateral view, with dorsal tip hook-shaped (Figs 4, 7). Lamella well-developed, with four projections: lateral one and posterior one long, lateral one with sharp, membranous end, posterior one with blunt end; anterior one broad, blunt; dorsal one short (Fig. 3). Terminal apophysis simple, broad and short, strongly membranous from prolateral view, with about one coil (Figs 2, 5). Embolus simple, perpendicularly curved at half length, with a sword-shaped end (Figs 4, 6).

###### Female:

**Figures 8–11.**
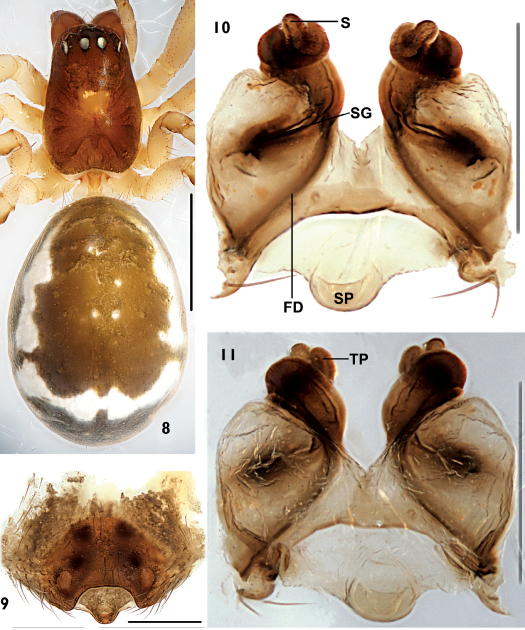
Neriene birmanica (Thorell, 1887), female from Nansan Town (Yunnan Province, China). **8** female habitus, dorsal view **9** epigynum, ventral view **10** epigynum without skin, ventral view **11** spermathecae, dorsal view. Scale = 1 mm (**8**), scales = 0.2 mm (**9**, **10**, **11**).

Total length: 2.86. Carapace: 1.14 long, 0.69 wide. Abdomen: 1.71 long, 1.30 wide. Carapace brown, unmodified. Eyes subequal. AER recurved, AME-AME shorter than AMEd, AME-ALE slightly shorter than AMEd; PER straight, PME-PME shorter than PMEd, PME-PLE about equal with PME-PME; ALE and PLE juxtaposed. Chelicerae brown, stridulatory ridges absent, promargin of fang groove with three teeth, median tooth largest; retromargin with three equal teeth. Lengths of legs: I 5.27 (1.44+1.61+1.44+0.78), II 4.60 (1.26+1.42+1.23+0.69), III 3.17 (0.93+0.94+0.79+0.51), IV 4.47 (1.37+1.27+1.21+0.62). Each tibia, patella and femur with two dorsal spines. Tm I: 0.22. Tm IV absent. Abdomen oval, without tubercle, the colour and patch see Fig. 8.

In ventral view, atrium opening small, trapeziform. Lateral depression present (Fig. 9). Scape arising from dorsal wall, short, with slightly rounded tip, and with small semi-covered depression on ventral surface (Fig. 10). Spermathecae as long as wide; spiral grooves started from entrances situated in the middle of the ventral wall of either atrium to the apical turning-points, with about two coils; fertilized ducts started from spermathecae, with (about) two coils; turning points situated laterally; spermathecae long and slender, situated laterally (Fig. 11).

##### Remarks.

Although we didn’t examine the type speciemens of Ambengana complexipalpis, the tiny paracymbium, the sword-shape embolic tip, broad and the short terminal apophysis, the uniquely trapeziform atrium opening shown in the original illustrations (Millidge and Russell-Smith 1992 figs 52–54) leave no doubts that our identification is correct. The original illustration of the spermathecae by Millidge and Russell-Smith (1992, fig. 55) is rather simplified and shows some differences with the specimen of Neriene birmanica we have examined (cf. Fig. 11). However, such the difference does not affect our identification.

##### Distribution:

**Figure 12:**
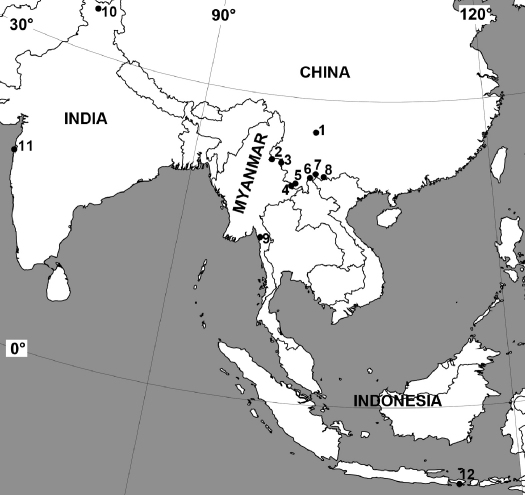
Distribution of Neriene birmanica (Thorell, 1887). Numbers correspond to the localities on the map: **1** China, Sichuan Province, Miyi County **2** China, Yunnan Province, Ruili City **3** China, Yunnan Province, Zhenkang County **4** China, Yunnan Province, Menghai County **5** China, Yunnan Province, Menghai County **6** China, Yunnan Province, Jiangcheng Hani and Yi Autonomous County **7** China, Yunnan Province, Luchun County **8** China, Yunnan Province, Jinping Miao, Yao, and Dai Autonomous County **9** Myanmar, Mawlamyine, Burma **10** Kashmir, Garhi near Jhelum **11** India, Bombay **12** Indonesia, Bali.

Southeast Asia (India, Kashmir, Myanmar, China, Indonesia) (Fig. 12).

## Supplementary Material

XML Treatment for Neriene
